# Multifunctional Nanosystems Powered Photodynamic Immunotherapy

**DOI:** 10.3389/fphar.2022.905078

**Published:** 2022-05-11

**Authors:** Yunong Ma, Fengfeng Xiao, Cuixia Lu, Liewei Wen

**Affiliations:** ^1^ Medical College, Guangxi University, Nanning, China; ^2^ Zhuhai Precision Medical Center, Zhuhai People’s Hospital (Zhuhai Hospital Affiliated With Jinan University), Jinan University, Zhuhai, China

**Keywords:** nanosystems, photosensitizers, photodynamic therapy, innate immunity, adaptive immunity

## Abstract

Photodynamic Therapy (PDT) with the intrinsic advantages including non-invasiveness, spatiotemporal selectivity, low side-effects, and immune activation ability has been clinically approved for the treatment of head and neck cancer, esophageal cancer, pancreatic cancer, prostate cancer, and esophageal squamous cell carcinoma. Nevertheless, the PDT is only a strategy for local control of primary tumor, that it is hard to remove the residual tumor cells and inhibit the tumor metastasis. Recently, various smart nanomedicine-based strategies are developed to overcome the barriers of traditional PDT including the drawbacks of traditional photosensitizers, limited tissue penetrability of light, inefficient induction of tumor cell death and tumor resistance to the therapy. More notably, a growing number of studies have focused on improving the therapeutic efficiency by eliciting host immune system with versatile nanoplatforms, which heralds a broader clinical application prospect of PDT in the future. Herein, the pathways of PDT induced-tumor destruction, especially the host immune response is summarized, and focusing on the recent progress of nanosystems-enhanced PDT through eliciting innate immunity and adaptive immunity. We expect it will provide some insights for conquering the drawbacks current PDT and expand the range of clinical application through this review.

## Introduction

**FIGURE 1:**
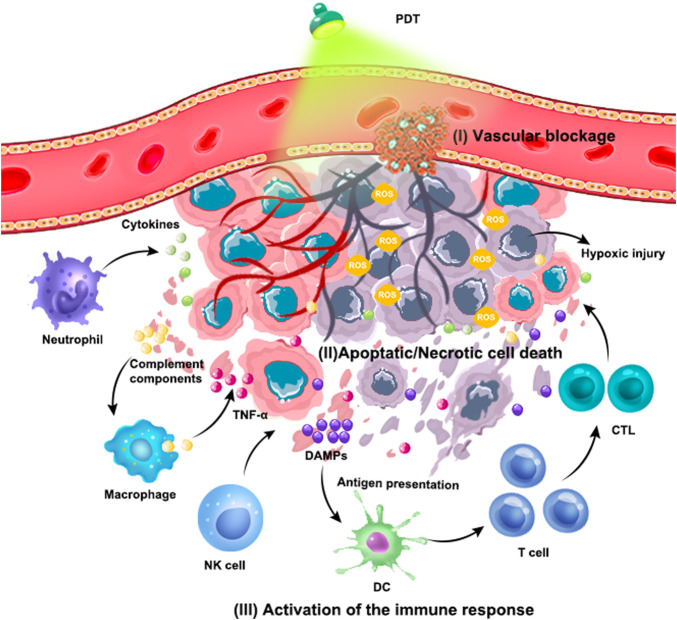
Schematic illustration of the antitumor mechanism of PDT.

Photodynamic Therapy (PDT) has been applied as adjuvant tumor therapy for more than 40 years (Li et al., 2020). It depends on a basic principles that the photosensitizer (PS) is excited by a light with an appropriate wavelength, and the excited PS directly transfers energy to the oxygen to produce reactive oxygen species (ROS), such as singlet oxygen (^1^O_2_), superoxide anions (O_2_^−^) and hydroxyl radicals (OH) in tumor cells (Dolmans et al., 2003). The highly reactive ROS will be result in the oxidation of the biomolecules in cells, including nucleic acids, lipids, and proteins, leading to severe alteration in cell signaling cascades or in gene expression regulation (Robertson et al., 2009). Consequently, the ROS directly or indirectly destroys tumor cells *via* apoptotic, necrotic and autophagy-associated cell death. Moreover, some photosensitizers are bound to serum protein and transfer to endothelial cells of tumor blood vessels. During laser irradiation, the tumor-associated vasculature will also be damaged, which can lead to thrombosis and hemorrhage in tumor blood vessels. Subsequently, the tumor growth can be inhibit owing to the lack of oxygen and nutrients (Janas et al., 2021; Wang et al., 2022c). In addition, PDT also induce the acute inflammation and trigger the release of cytokines and stress response proteins. Firstly, as a major characteristic of acute inflammation, the neutrophils will be activated in circulation and migrate across blood vessels to move to the infectious or injured sites (Chu et al., 2018), thus the acute inflammation induced by PDT can induce the recruitment of some neutrophils to kill cancer cells. Meanwhile, the injuries of the blood vessel and tumor cells also attract the macrophages infiltration, regulating the polarization of macrophage and improving the phagocytosis of macrophages to tumor cells (Zhou et al., 2009; Korbelik and Hamblin, 2015). Several studies have also suggested that PDT can improve the Natural Killer (NK) cells activity and initiate the immunity. Apart from the direct cytotoxicity, NK can stimulate the maturation of dendritic cells (DCs) and subsequently activate the adaptive immune cells like monocytes, cytotoxic T lymphocytes (CTLs), and B cells to enhance the whole immune response by secreting cytokines (Cantoni et al., 2016; Cerwenka and Lanier, 2016; Wang et al., 2019b). Notably, the PDT will trigger the release of adenosine triphosphate (ATP) and high-mobility group box 1 protein (HMGB1) from the dying cells, meanwhile, the calreticulin (CRT), heat shock proteins (HSPs) will be exposed on the surface of dying cells. These molecular provide “eat me” signal to initiate an immune response (Shaif-Muthana et al., 2000). It promotes the mutation of antigen presenting cells (APCs) and presentation of tumor associate antigen (TAA). Meanwhile, the secretion of cytokines (IFN-γ, IFN-α) will be boosted and further promote the APCs maturation and CTLs homing (Figure 1) (Beltran Hernandez et al., 2020). The activation of anti-tumor immunity not only contributes to the destruction of primary tumor cells, but also destroys the tumor cells even at isolated locations (Ji et al., 2022).

The mechanisms of tumor damage caused by PDT mainly include the following types: 1) PS is enriched in tumor vascular endothelial cells, causing platelet coagulation, vasoconstriction, and thrombosis. Tumor blood vessels are blocked, resulting in hypoxic infarction of tumor tissue. 2) ROS directly destroys proteins, nucleic acids, lipids, etc. of cells, causing apoptosis, necrosis and autophagy of cells. 3) PDT-triggered inflammatory responses activate innate and adaptive immune responses.

Nevertheless, the broad application of conventional PDT remains many challenges in the clinic practice. It is well-known that PS, oxygen, and light as three essential components are involved in PDT. Typically, most of the PS lacks the tumor-targeting ability and the effective dosage is insufficient in tumors (Idris et al., 2015). Furthermore, the hypoxia tumor microenvironment, limited light penetration towards tumor tissues, and insufficient T cell infiltration in tumors also greatly limited the efficacy of PDT (Zhang et al., 2018a). Recently, with the development of nanotechnology, various advanced nanoparticles were developed to overcome above drawbacks and expand the range of application, showing the great potential for improving the PDT efficacy. Numerous nanomaterials have been reported to have the ability to produce ROS under the light excitation, enabling them to serve as nano-photosensitizers in PDT (Table 1) (Wang et al., 2015; Sun et al., 2019b). Moreover, some nanoparticles are also ideal carriers for traditional photosensitizers (Lucky et al., 2015; Xie et al., 2021). The nano-photosensitizers and nano-delivery systems are able to accumulate more efficiently in tumor tissue through the enhanced permeability and retention (EPR) effect owing to the particle size and the abnormal vascular structure of tumors (Peer et al., 2007; Hao et al., 2020; Li et al., 2021b; Liang et al., 2021b). The nanomaterials can also be further modified or functionalized to improve the targeting efficacy, enhancing the dosage of in tumor and reducing toxicity to normal tissues (Zhong et al., 2015; Seidi et al., 2018; Liang et al., 2021a). Furthermore, nanomaterial-based photosensitizers have better photostability than traditional ones, which is able to produce ROS for a longer time (Wang et al., 2020b). It is worth noting that nanomaterials mediated PDT endow the excitation light source and approach more flexible and adjustable. For example, various lanthanide-doped upconversion nanoparticles (UCNPs) are developed and applied for UCNPs-based PDT. It will be excited by near infrared light (NIR), which can penetrate deep tissues (>3 cm) with lower light scattering and absorption of human tissues (Chen et al., 2017; Zhang et al., 2018a). Other excitation approach including ultrasound and X-ray gradually derived new therapeutic strategies termed sonodynamic therapy (SDT) and radiodynamic therapy (RDT), respectively, also provide novel approach for breaking through the limitation of tissue penetration depth (Chen et al., 2015; Lu et al., 2018; Pan et al., 2018; Ni et al., 2022; Nowak et al., 2022). To relieve the hypoxia in tumor microenvironment and improve the PDT efficacy, several strategies based on nanomaterials have emerged so far. In addition to the direct delivery of oxygen with nanoplatform, some nanoparticles can be served as nanoreactor to catalyze the release of oxygen in tumor microenvironment, such as MnO_2_, CaO_2_ and two-dimensional nanomaterials (Fan et al., 2016; Ji et al., 2019; Zhou et al., 2020; Wang et al., 2022a; Jin et al., 2022; Zeng et al., 2022). Besides, acceleration of intratumoral blood flow is also useful for increasing O_2_ concentrations in hypoxic tumors. Previous research suggests that photothermal-mediated heating will increase the blood flow and combination with photothermal therapy is regarded to enhance PDT inhibition of hypoxic tumors (Wu et al., 2019a; Chen et al., 2022). Benefiting from the unique characteristics of multifunctional nanomaterials, multiple therapeutic strategies can be simultaneously mediated by one nanoplatform to synergistically inhibit tumor, such as the combination of gas therapy (Wan et al., 2018; Wang et al., 2022b) and chemotherapy (Yang et al., 2019a; Wang et al., 2019e), etc., Recently, some emerging nanomaterials such as heterojunction-based nanoparticles have been prepared to enhance the generation efficacy of ROS through catalytic reaction, deriving a new therapeutic strategy termed catalytic therapy. The combination of PDT and catalytic therapy implied a promising strategy to strengthen the therapeutic efficiency (Pan et al., 2019; Kang et al., 2021; Kong et al., 2021; Pan et al., 2022).

**TABLE 1:** Summary of nanocomposites containing photosensitizers for cancer therapy.

Photosensitizer	Nanoplatforms	Wavelength	Cancer	References
Porphyrin sodium (Photofrin)	Metal-organic frameworks (MOFs)	630 nm	Breast cancer, cervical cancer	Choi et al. (2018), Marydasan et al. (2019)
5-aminolevulinic acid (5-ALA)	5-ALA-SQ NPs, nanogels, ALA-OHex micelles	630 nm, 660 nm	Prostate cancer, breast cancer, cervical cancer	Babic et al. (2018), Wang et al. (2020a), Li et al. (2021a)
Chlorin e6 (Ce6)	rGO-PEG/Ce6 NPs, PEG-Ce6-Gd NPs, Uccinate (TPGS)–IR820/Ce6 micelles	630 nm, 660 nm, 808 nm	Breast cancer, glioma, melanoma	Hu et al. (2018), Lee et al. (2020), Xu et al. (2021)
Rose Bengal (RB)	Mesoporous silica NPs, RB-loaded peptido-nanomicelles (RBNs)	532 nm, 585 nm	Glioma, squamous cell carcinoma	Sun et al. (2019a), Zhang et al. (2019)
Indocyanine green (ICG)	Holo-Tf-indocyanine green (holo-Tf-ICG) NPs, folate decorated polymeric micelles (FA Co-PMs), DOX/ICG (DI) micellar	808 nm	Breast cancer, glioma, liver cancer	Zhu et al. (2017), Zhang et al. (2018b), Chen et al. (2019)
Infrared 780 iodide (IR780)	Polydopamine nanoclustered micelles, IR780-DOX-PEG NPs	808 nm	Breast cancer	Yang et al. (2019a), Xing et al. (2019)
Infrared 820 (IR820)	IR820 1-methyl-tryptophan (IR820-1 MT) NPs, zinc protoporphyrin (ZnPP) conjugated micelles	808 nm	Breast cancer, melanoma, lung cancer	Noh et al. (2018), Zaharie-Butucel et al. (2019)
Infrared 806 (IR806)	Metal-organic frameworks (MOFs), IR806 chitosan liposomes	793 nm, 980 nm	Breast cancer, cervical cancer	Deng et al. (2017), Lin et al. (2018); Jogdand et al. (2020)

Tumor recurrence and metastasis are vital causes of treatment failure and death of patients. Although PDT can induce host anti-tumor immune response, the short-acting and insufficient anti-tumor immunity is hard to control the tumor recurrence and metastasis due to the existence of immunosuppressive microenvironment. A large number of studies currently is committed to elicit robust anti-tumor immunity after PDT, and imply that activation of antitumor immune effects is a highly promising strategy to enhance PDT efficacy and expand PDT indications. Nowadays, the advanced nanomaterials have opened new promising avenues to refine and improve the anti-tumor efficacy through combining PDT with immunity activation. In addition to improve the therapeutic efficacy by excellently codelivering PS to tumors, relieve hypoxic microenvironment and combining with other treatment approaches, etc., the multifunctional nanomaterials can regulate the tumor immune microenvironment, including eliciting innate immunity and adaptive immunity (Table 2) (Xu et al., 2017; Sang et al., 2019; Chen et al., 2020; Chen et al., 2021).

**TABLE 2:** Summary of photodynamic therapy and immunotherapy combinatorial treatments.

Immunity effect	Nanoplatform	Cancer	Methods	References
Activate NK cells	Liposomes	Melanoma	Loaded with NK cell agonist	Deng et al. (2018), Wang et al. (2019b)
Induce M1 macrophage polarization	Liposomes; metal-organic frameworks (MOFs)	Breast cancer	Nanoparticles wrapped with TAMs/ neutrophil/NK cell membranes; nanoparticles repolarize macrophages	Deng et al. (2018), Wang et al. (2019c), Jiang et al. (2020), Chen et al. (2021), Qiu et al. (2021)
Activate DCs and increase DCs antigen presentation	Nanocapsule; liposome	Gastric carcinoma	Nanoparticles loaded with DCs agonists	Im et al. (2019), Mao et al. (2020)
Activate cytotoxic T lymphocytes and deplete Tregs	Liposome; micelle	Breast cancer	Loaded with drugs against immunosuppressive cells	Cheng et al. (2021), Ding et al. (2021)
Blockade immune checkpoint	Metal-organic frameworks (MOFs); micelle; liposome	Breast cancer; melanoma; bladder cancer; melanoma	Co-administration with CTLA-4 and PD-L1; Or co-delivering the IDO-1 inhibitor	Wang et al. (2016), Gao et al. (2020), Yao et al. (2020), Choi et al. (2021), Zhou et al. (2021)

## Eliciting Innate Immunity

After PDT, cell debris or various cytoplasmic components resulting from tumor death and lysis can cause a strong inflammatory response in surrounding tissues. Various cells in the TME, such as surviving tumor cells, damaged endothelial cells, tumor stromal cells, etc., can release numerous pro-inflammatory mediators, including arachidonic acid, cytokines such as MIP2 (CXCL2), IL6, IL- 1β, TNFα, complement system, etc.. These components enhance chemotaxis, activation, and phagocytosis of macrophages, and aggregate neutrophils, triggering a strong innate immune response. At the same time, macrophages will present cell debris and dead tumor cells as antigens to T lymphocytes, and correspondingly activate lymphocyte-based adaptive immunity (Jin et al., 2022). Therefore, PDT-induced local tumor tissue inflammatory response activates innate and adaptive immune responses (Beltran Hernandez et al., 2020). The innate immune cells can detect tumors, induce and amplify adaptive immune responses, and exert direct effector responses. Given the crucial role of innate immune responses in antitumor immunity, harnessing manipulates innate immune responses in cancer opens up new possibilities for long-lasting, multilayered tumor control.

### Potentialities of Neutrophils to Enhance Photodynamic Therapy

Neutrophils, known as polymorphonuclear leukocytes (PMN), are the major immune cell population in human blood and serve as the first line of defense against invading pathogens (Rosales, 2018). In recent years, increasing evidence has shown that neutrophils have host defense, immunomodulatory functions, and play an important role in cancer therapy (Wu et al., 2019b). The polarization of primary human neutrophils *in vitro* to generate N1 and N2 neutrophils was first proposed by Mareike et als. N1 polarized neutrophils have pro-inflammatory properties and promote the release of interferons, chemokines, and tumor necrosis factors (Ohms et al., 2020). Although there is an urgent need to fully understand the interaction between neutrophils and their tumor microenvironment, most of the current studies are still limited to mouse tumor models, and continued research is needed to verify whether human neutrophils have macrophage-like polarization (Eruslanov et al., 2017; Wu et al., 2020).

Numbers studies have shown that PDT promotes neutrophil infiltration in tumor tissues and then activates innate immunity (Cecic et al., 2006). Since neutrophils are able to migrate into inflamed tumors, the use of neutrophil-mediated drug delivery systems to enhance drug accumulation and sustained release at tumor sites opens new avenues for nanomedicine development. Therefore, Qiu et al. (2021) constructed a nanocomplex (SA-2@NCs) capable of targeting activated peripheral blood neutrophils (PBN), which was formed by encapsulating ibrutinib (IBR) drugs with targeted sialic acid (SA). First, liposomes loaded with photosensitizer DIR were injected into the tail vein, and PDT/PTT treatment was performed to induce acute inflammation and rapidly activate PBNs to infiltrate the tumor site. Then, SA-2@NCs nanocomplexes were injected into the tail vein, which accumulated at the tumor site after being internalized by activated PBN. Combining IBR-mediated immunotherapy and DIR-mediated PDT/PTT for antitumor can effectively inhibit tumor growth and metastasis (Qiu et al., 2021). However, there are some difficulties in the extraction and preservation of neutrophil membranes, and the stability of nanoparticles needs to be improved.

In summary, nanoparticles wrapped with neutrophil membranes promote the recruitment of nanoparticles in tumor tissues and improve the tumor targeting of nanoparticles. Notably, nanoparticles loaded with immune activators can be tried to activate neutrophil N1 polarization and enhance antitumor immune response.

### Activation of Natural Killer Cells

**FIGURE 2:**
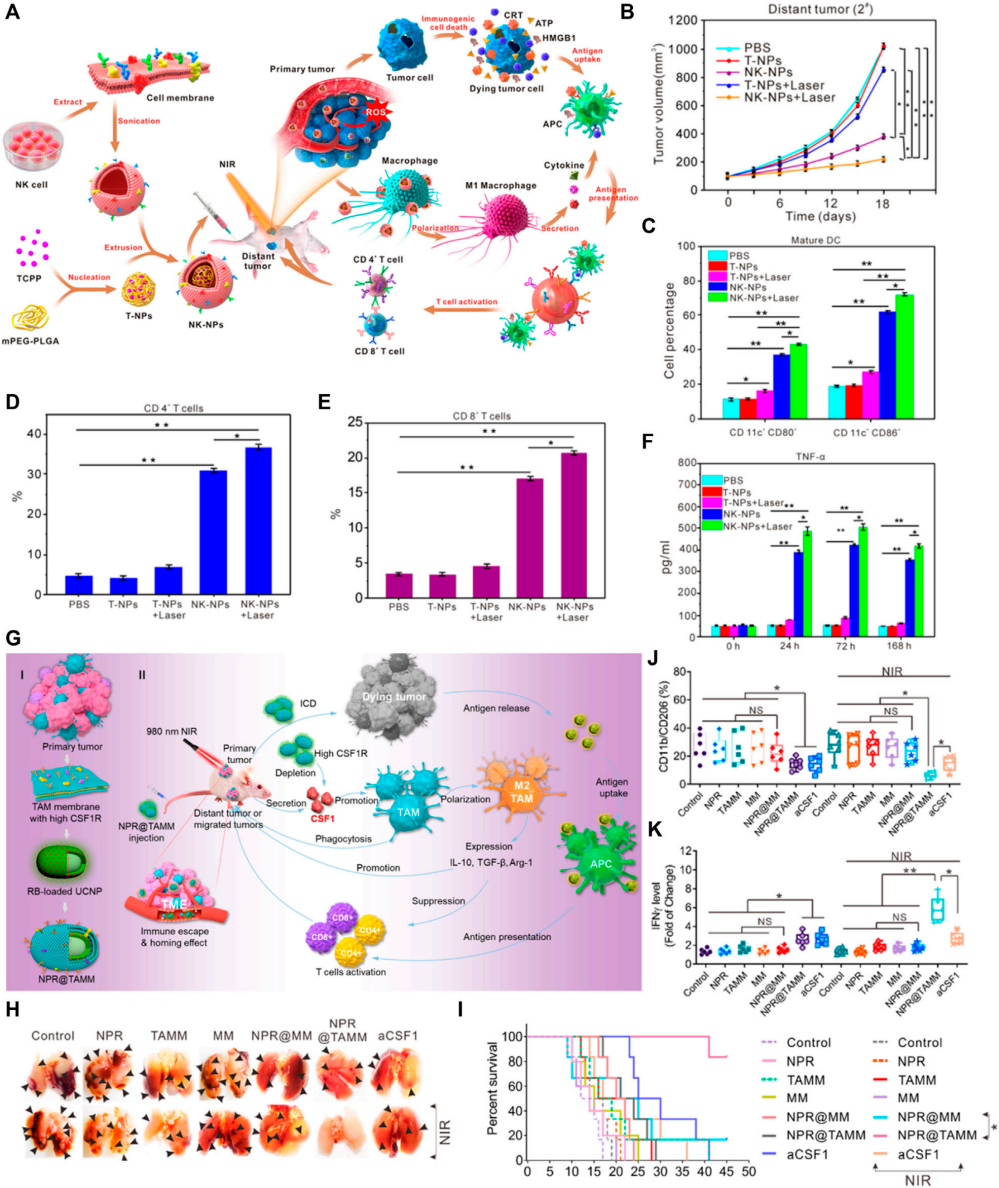
Example of nanomedicine-based PDT to activate innate immune responses. **(A)** Schematic Illustration of NK Cell-Membranes-Cloaked Nanoparticles for PDT-Enhanced Cell-Membrane Immunotherapy. **(B)** Growth curves for the distal tumors. **(C)** *In vivo* maturation of DCs (CD80^+^ and CD86^+^) from tumor-draining lymph nodes in BALB/c mice following intravenous injection of T-NPs or murine NK-NPs (*n* = 3 per group). **(D)** Proportions of tumor-infiltrating CD4^+^ T cells. **(E)** Proportions of tumor-infiltrating CD8^+^ T cells. **(F)** Pro-inflammatory cytokines (TNF-α) levels in the sera of mice treated with murine NK-NPs-mediated PDT from day 0, day 1, day 3, and day 7 (Deng et al., 2018). **(G)** Schematic illustration of the tumor-associated-macrophage-membrane-coated upconversion nanoparticles for improved photodynamic immunotherapy. **(H)** Photographs show representative external views of lung nodules. **(I)** The survival curve of tumor-bearing mice calculated by Kaplan−Meier estimate. **(J)** Quantification by flow cytometry of the ratio of CD11b + CD206 + cell populations in the different treatment groups of tumor-bearing mice. **(K)** ELISA assay of IFN-γ in tumor-bearing mice with different treatments (Chen et al., 2021). Data are means ± SD. *p < 0.05; **p < 0.01. NS, no significance.

NK cells are innate immune cells that are the first line of defense against infection and cancer. NK cells can spontaneously damage target cells in the absence of antigen-specific stimulation and major histocompatibility complex. Numerous studies have shown that the antitumor immune response induced by PDT enhances the activity of NK cells. Enhancing PDT-induced immune response by activating NK cells may be benefit improve the therapeutic effects (Zheng et al., 2013). Protective mechanisms such as anti-apoptotic mechanisms and therapeutic escape pathways in melanoma hinder the application of PDT in melanoma. Therefore, Wang et al. (2019b) designed a special nanoliposomes to enhance Melanoma PDT and immune activation of NK cells. The main components of nanoliposomes, including phosphatidylcholines, Ce6, and low molecular citrus pectin (LCP), are all in clinical use or biodegradable. Nanoliposomes as a trigger to enable the cytoplasmic release of LCP. LCP promotes the binding of NKG2D to MICA on tumor cell membranes and indirectly activates NK cells, giving the nanoparticles a synergistic effect of enhancing PDT and NK cell-related immunity. However, further more molecular and immune studies should be performed in the future to elucidate the role of LCP in PDT antitumor immunity. Furthermore, the natural tropism of NK cells is exploited during PDT to improve the tumor-targeting effect of nanoparticles. In antitumor immunotherapy, NK cell membrane can induce polarization of M1-macrophages, providing membrane immune-inducers for stimulating the immune response during tumor therapy. Therefore, some researchers have designed a NK cell membrane-encapsulated nanoparticle (NK-NPs), which target cancer cells and induce M1 macrophage polarization, thus induce tumor-specific immune responses (Figure 2A) (Deng et al., 2018). The *in vivo* results demonstrated that NK-NPs-mediated PDT could enhance NK cell membrane immunotherapy, eliminate primary tumor growth, and produce distant effects that inhibit distant untreated tumors. As shown in Figures 2B–F. In addition, blockade the immune checkpoint of NK cells may enhance the therapeutic effect of PDT. For example, some researchers have found that T-cell immunoglobulin and ITIM domain (TIGIT) is expressed on activated T cells and is also found on NK cells, memory T cells, a subset of Treg cells as well as follicular T helper cells. TIGIT directly inhibits NK and effector T cells, and it also inhibits immune responses through promoting Treg cell function (Anderson et al., 2016). In some mouse models, blocking TIGIT prevents NK cell depletion. And blocking T-cell immune receptor with TIGIT enhances NK cell-dependent tumor-specific T-cell immunity. Loading TIGIT inhibitors into nanoparticles may be a new direction for future research.

In conclusion, the immune response can be improved by regulating the activation of NK cells at different targets during PDT. The natural tropism of NK cell membranes is exploited to improve the targeting of nanoparticles to tumors. The immunotherapeutic approach of the NK synergistic PDT offers a strategy for tumor immunotherapy.

### Regulation of Macrophage Polarization

It is now well established from a variety of studies that macrophages can be divided into M1 macrophages, which have antitumor effects, and M2 macrophages, which promote tumor proliferation (Qian and Pollard, 2010; Lu et al., 2016). *In vivo*, M1 macrophages are responsible for recruiting Helper T cells, cytotoxic T lymphocyte, and NK cells, that directly target infected or tumor cells (Mantovani et al., 2004; Beatty et al., 2011; Murray and Wynn, 2011; Butcher and Galkina, 2012). Compared with M1 macrophages, M2 macrophages exhibit an anti-inflammatory phenotype and are present in infections, allergies, tissue reconstruction, and tumor development (Satoh et al., 2010). Tumor-associated macrophages (TAMs) are a prominent component of the stroma and leukocyte infiltrates in tumors (Azria et al., 2003; Balkwill, 2009). On the one hand, TAMs surround tumor cells and interact in the TME to promote tumor growth, progression, and metastasis, leading to immunosuppression. On the other hand, TAMs inhibit tumor growth after transformation to the M1 phenotype (Qiu et al., 2018). Therefore, a large number of studies support the concept of TAMs reprogramming as a sufficient and feasible approach to initiate T-cell-mediated antitumor immunity (Cassetta and Pollard, 2018). PDT induce immunogenic cell death (ICD) to release damage-associated molecular patterns (DAMPs), thus polarize macrophages from an immunosuppressive M2 phenotype to an antitumor M1 phenotype (Tan et al., 2016; Zhou et al., 2018). A large number of studies have confirmed demonstrated that the polarization of M1 macrophages could be promoted by structural modification of PDT nanoparticles and the loading of photosensitizer (Wang et al., 2019c). In addition, some nanoparticles also affect macrophage polarization by initiating ferroptosis. Ferroptosis is a new cell death format identified in recent years. Ferroptosis can effectively inhibit tumor growth and induce immune response. Considering the central role of iron in ferroptosis, many studies have focused on iron-based nanomaterials. Jiang et al. (2020) reported a PLT membrane-camouflaged Fe_3_O_4_-SAS [magnetic nanoparticle loaded with sulfasalazine (SAS)]. Fe_3_O_4_ nanoparticles are ferroptosis inducers. And the study revealed that Fe_3_O_4_-SAS@PLT-mediated ferroptosis could repolarize macrophages from immunosuppressive M2 phenotype to antitumor M1 phenotype and elicit an effective immune response. This nanoparticle-mediated ferroptosis combined with SAS immunotherapy effectively inhibited tumor growth and metastasis compared to a single treatment (Jiang et al., 2020). On the other hand, Chen et al. designed a PDT nanoparticle (NPR@TAMMs) wrapped by TAMs membrane (TAMM). Studies on the TAMM encapsulated nanoparticles showed unique antigen-homing affinity and biocompatibility. Meanwhile, TAMMs can deplete macrophage colony-stimulating factor 1 (CSF1). CSF1 is a key regulator of monocyte/macrophage differentiation and maintains the tumor-promoting function of TAMs. By displaying TAM membranes on the surface of nanoparticles, it is expected that NPR@TAMMs can mimic the source cells, thus binding to the immunomodulatory molecule CSF1, reducing serum CSF1 levels and blocking CSF1/CSF1R signaling, leading to immunosuppressive inactivation (Figures 2G–I). This PDT-immunotherapy approach based on macrophage membrane provides a new strategy for personalized cancer treatment (Chen et al., 2021). Overall, the PDT nanoparticles could improve the antitumor innate immune response through loading of agonists or modifying with TAMM and NK cell membranes.

## Eliciting Adaptive Immunity

A growing number of studies have shown that acute inflammation triggered by PDT recruits leukocytes to the tumor area and secretes chemokines, then promote the maturation of dendritic cells (DCs) and activating T and B cells, leading to adaptive immune responses (Brackett and Gollnick, 2011; Yang et al., 2016). Although some studies have suggested that B cells play a role in PDT-induced antitumor immunity and mechanism of B cell-mediated antitumor immunity has not been elucidated (Preise et al., 2009; Brackett and Gollnick, 2011; Rocha et al., 2015). Therefore, in this section, we focus on PDT-induced T-cell-mediated tumor-specific immune responses.

### Promotion of Dendritic Cells Maturation

Currently, the mechanism of antitumor immune response by PDT has been described mainly by the immunogenic cell death (ICD), which can transform a “cold” tumor into a “hot” (Kumar et al., 2021). ICD induces DAMP such as calreticulin (CRT), high mobility group protein 1 (HMGB1), and heat shock protein (HSP-70/90) (Adkins et al., 2014; Radogna and Diederich, 2018; Li et al., 2019). These danger signals cause APC to present antigens (Turubanova et al., 2019). Subsequently, T lymphocyte infiltration increases and its mediated adaptive immune response is activated (Kabingu et al., 2007; Duan et al., 2016). Consequently, activation of DC maturation and promotion of antigen presentation may contribute to the improvement of the antitumor efficacy of PDT.

**FIGURE 3:**
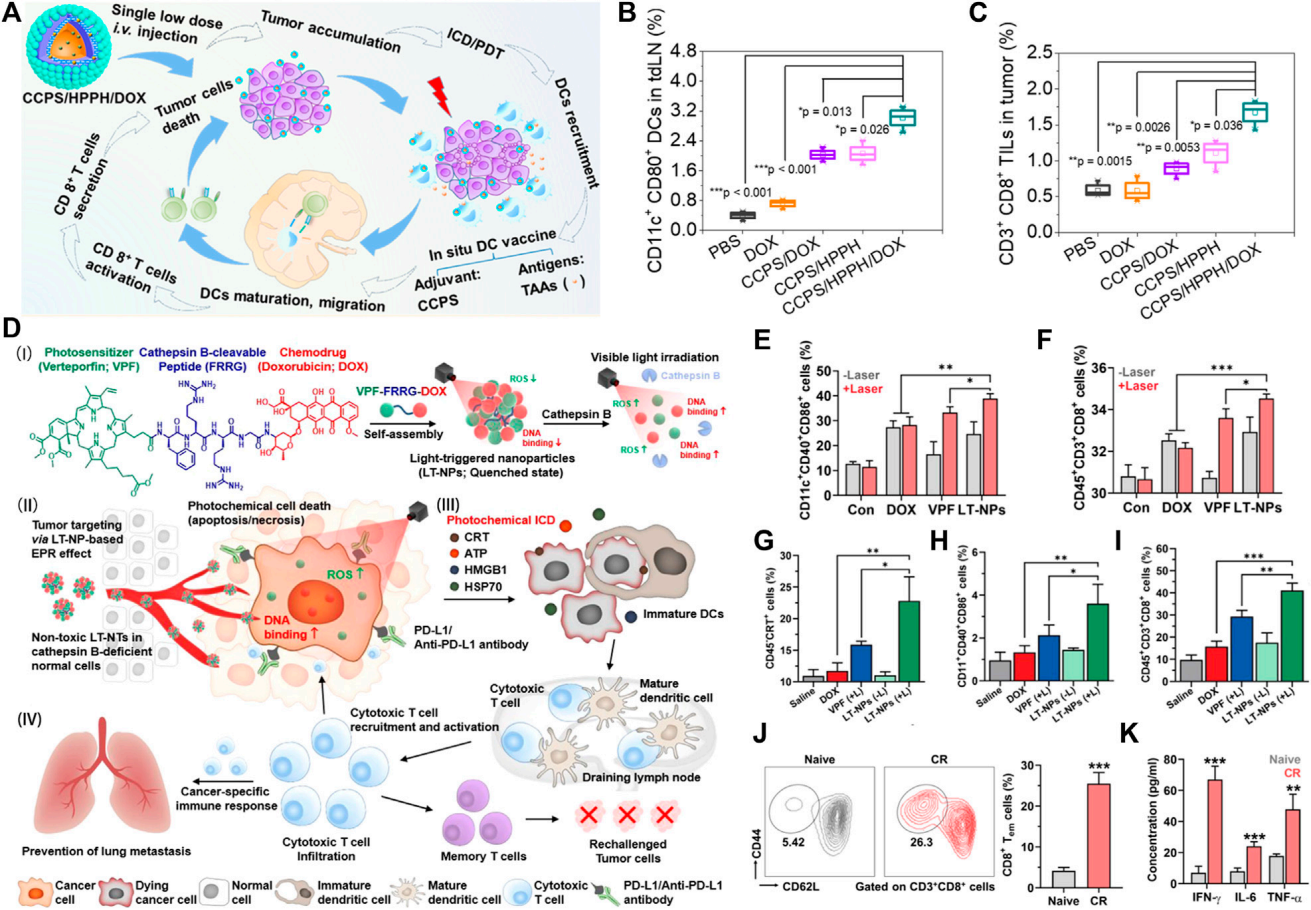
Example of nanomedicine-based PDT to activate adaptive immune responses. **(A)** Schematic illustration of an *in situ* DC vaccine exploiting chimeric cross-linked polymersomes (CCPS) as adjuvant combined with tumor-associated antigens (TAAs) induced by PDT and ICD for MC38 colorectal cancer immunotherapy. **(B)** Activated DC ratio in tumor-draining lymph nodes (tdLNs) for mice treated with different nanoformulations (*n* = 3). **(C)** Tumor infiltrating lymphocytes in tumor sites after treatment (*n* = 3) (Yang et al., 2019b). **(D)** Schematic illustration of visible-light-triggered prodrug nanoparticles (LT-NPs) combined chemotherapy and PDT to potentiate checkpoint blockade cancer immunotherapy. **(E,F)** Percentage of **(E)** matured DCs (CD11c + CD40 ^+^ CD86^+^) and **(F)** cytotoxic T cells (CD45 ^+^ CD3^+^ CD8^+^) in lymphocytes after coculture with culture medium containing CT26 cells treated with DOX, VPF, or LT-NPs in the presence or absence of light irradiation (*n* = 5). **(G–I)** Percentage of **(G)** CRT-positive cancer cells (CD45−CRT+), **(H)** tumor-infiltrating matured dendritic cells (CD11c + CD40 ^+^ CD86^+^), and **(I)** tumor-infiltrating cytotoxic T cells (CD45 ^+^ CD3^+^ CD8^+^) on day 7 after treatments (*n* = 5). **(J)** Percentage of splenic effector/memory T cells among the CD8^+^ T cells (CD3^+^CD8^+^CD44 ^+^ CD62L^low^) in mice that experienced CR by LT-NPs (+L) with anti-PD-L1 antibody on day 100 after treatment and naive mice (*n* = 5). **(K)** Cytokine levels in serum isolated 20 days after CR mice were rechallenged with secondary tumors, compared to naive mice (*n* = 5) (Choi et al., 2021). Data are means ± SD. *p < 0.05, **p < 0.01, ***p < 0.001.

Numerous studies have reported enhanced DC maturation and activation and increased secretion of pro-inflammatory cytokines after PDT (Gollnick and Brackett, 2010; Kushibiki et al., 2010; Brackett and Gollnick, 2011; Zheng et al., 2016). The infiltration of DCs in tumor tissues is essential for antigen presentation in the immune response. On the one hand, adoptive DCs can be attempted to promote the immune response. Sur et al. (2008) appropriately transferred DCs of homozygous bone marrow origin into PDT-treated tumors and found that this treatment improved the survival rate of rats. On the other hand, designing DCs vaccines for tumor-targeted immunotherapy. Studies have shown that a DC-based tumor vaccine would be a safe and promising tool for cancer therapy. Yang et al. (2019b) reported a multimeric nanoformulation capable of producing tumor-associated antigens (TAAs) and acting as DC immune adjuvants. The nanoformulation was based on a chimeric cross-linked polymersome (CCP) as a carrier to encapsulate doxorubicin hydrochloride (DOX) and a photosensitizer 2-(1-hexyloxyethyl)-2-devinyl pyropheophorbide-a (HPPH). The chemotherapeutic drug DOX can induce immunogenic cell death (ICD), the cell debris generated by PDT acts as TAAs, and in addition, CCPS with amine groups acts as an adjuvant for DCs. Combined PDT and immune adjuvants can enhance the recruitment of TAAs and DCs, promote the maturation of DC cells, and trigger a cascade of immune responses (Figure 3A) (Yang et al., 2019b). About 3.02% of the DCs were activated in the tumor tissue of the CCPS/HPPH/DOX treated group and about 1.7% of the cytotoxic T lymphocyte population infiltrated into the mouse tumor tissue, which was significantly higher than that of the control group (Figures 3B,C). Garg et al. (2016) developed a DC-based vaccine using chrysin-PDT that induced a strong immune response and improved overall survival in a mouse glioma model. Numerous trials have shown that dendritic cell-based vaccination can induce tumor-specific T-cell responses. However, the therapeutic effect of activating DCs to promote antigen presentation is limited due to the lack of prevalent antigens in the immune system. There are several other studies that focus on immune regulation during PDT. A novel therapeutic strategy is to construct the nanoparticles loaded with photosensitizers and DCs agonists. Im et al. (2019) reported a low oxygen response mesoporous silica nanocapsule (CAGE) for PDT. The nanocapsules were loaded with Ce6 and their surfaces were modified with glycol chitosan (GC) and PEG. The negatively charged CpG is loaded onto the CAGE surface by electrostatic interaction with GC. CpG oligonucleotides (CpG) bind to TLR9 and activate dendritic cells, upregulating costimulatory markers for antigen presentation. In this study, modification of nanoparticles activated DCs and increased DCs antigen presentation, which subsequently enhanced antigen presentation (Im et al., 2019). The developed immune adjuvant delivery system reflects its function in the tumor-associated immune microenvironment, demonstrating the potential of adjuvant immunotherapy combined with PDT in antitumor therapy.

In sum up, activating DC to enhance the antitumor immune efficacy of PDT is a promising approach. Adoptive DC, DC vaccine and the DCs agonists could promote DC maturation, thus facilitate antigen presentation and subsequent immune response.

### Depletion of Tumor-Associated T Regulatory Cells

Previous studies have shown that PDT induces adaptive immune responses in a CTL-dependent manner (Zheng et al., 2013; Kleinovink et al., 2017). An important aspect of PDT-mediated antitumor immunity is the effective infiltration of T cells into tumors (Ino et al., 2013). Clinical studies also have shown that PDT activates CD4^+^ helper T cells (Hou et al., 2020). And a number of experiments have demonstrated that patients with vulvar intraepithelial neoplasia who responded to PDT had increased levels of infiltrating CD8^+^ T cells after treatment. However, the tumor tissue makes use of all kinds of immunosuppressive mechanisms to establish an immunosuppressive microenvironment to resist and suppress antitumor immune response. The presence of immune checkpoint molecules and tumor-infiltrating immunosuppressive cells in tumor tissue limits the activation and effective functioning of antigen-reactive T cells and enables tumors to escape from immune elimination.

Numerous tumor-associated T regulatory cells (Tregs) inhibit the activation and expansion of tumor antigen-specific effector T cells, which provides a favorable environment for tumor growth (Nishikawa and Sakaguchi, 2014). Depletion of tumor-infiltrating Tregs would be beneficial to trigger antitumor immune response. Remarkably, systemic depletion of Tregs may induce severe autoimmune diseases and hyperimmune responses (Wing and Sakaguchi, 2010). CD25-targeted PDT as reported by Oh et al. (2017) can induce Tregs apoptosis in tumors and inhibit tumor growth. It is well known that immunosuppressive cells, including myeloid-derived suppressive cells (MDSCs), M2-like tumor-associated macrophages (TAMs), and regulatory T cells (Tregs), are responsible for tumor immune escape. Loading drugs against immunosuppressive cells into nanoparticles can down-regulate the activity of immunosuppressive cells, providing a new path for promoting PDT antitumor immunotherapy. Ding et al. (2021) prepared a liposome (LIC) composed of phosphoinositol 3-kinase (PI3Kγ) inhibitor IPI-549 and photosensitizer Chlore6 (Ce6). IPI-549 is a selective PI3Kγ inhibitor that inhibits PI3Kγ in MDSCs, leading to downregulation of arginase 1 (Arg-1) and ROS, promoting apoptosis of MDSCs and reducing their immunosuppressive activity against CD8+T cells. It promotes the proliferation and activation of cytotoxic T lymphocytes (CTL), reduces Tregs, and repolarizes TAMs to an M1-like phenotype (Ding et al., 2021).

In addition to Tregs, the other natural immunosuppressive cells including MDSCs and M2 macrophages are also attracted to the tumor microenvironment. Thus, it would be highly desirable to develop anti-immunosuppressive cells-based therapies to achieve therapeutic feasibility.

### The Combination of Photodynamic Therapy With Immune Checkpoint Blockade

Immune checkpoints are a plethora of inhibitory pathways present in the immune system initiated by ligand-receptor interactions that regulate immune responses in surrounding tissues (Pardoll, 2012). Immunosuppression is mediated by cytotoxic T lymphocyte-associated antigen-4 (CTLA-4) and programmed death-1 (PD-1). These two immunomodulatory receptors are expressed on T cells (Quezada et al., 2011; Pardoll, 2012). T-cell receptors (TCR) initiate an immune response by recognizing antigens. This is regulated by the balance between co-stimulatory and inhibitory signals (i.e., immune checkpoints) (Zou and Chen, 2008). Immune checkpoints are easily blocked by antibodies or regulated by recombinant forms of ligands or receptors, preventing antitumor immunity. Combining immune checkpoint inhibitors with other therapies including PDT may improve the effectiveness of antitumor treatment (He et al., 2016; Wang et al., 2016; Yang et al., 2017; Yuan et al., 2021).

CTLA-4 is the first clinically targeted immune checkpoint receptor that is expressed exclusively on T cells, where it primarily regulates the early stages of T-cell activation. CTLA-4 neutralizes the activity of the T-cell co-stimulatory receptor CD28 (Lenschow et al., 1996). Once antigen recognition occurs, CD28 signaling amplifies TCR signaling to activate T cells (Schneider et al., 2006). CTLA-4 blockade modulates immunosuppression within tumors and has been approved by the US Food and Drug Administration for the treatment of several types of cancer therapies (Wang et al., 2016; Xu et al., 2017). Wang et al. (2019b) reported a nanoparticle UCNP-CE6-R837, which promoted CTLA-4 checkpoint blockade by loading immune adjuvant, effectively inhibited the immunosuppressive activity of Treg cells, increased the ratio of CTL to Treg cells, and promoted antitumor cell immunity. Surprisingly, PDT based on UCNP-CE6-R837 combined with CTLA-4 blockade can effectively induce the generation of immune memory response to prevent tumor recurrence, similar to the function of cancer vaccine. This study proved that PDT combined with cancer immunotherapy can achieve significant synergistic therapeutic effects (Wang et al., 2019b).

In contrast to CTLA-4, PD-1 has a remarkably promising immune checkpoint receptor, and has the primary role of regulating effector T-cell activity within tissues and tumors and limiting autoimmunity (Keir et al., 2006; Okazaki and Honjo, 2007; Keir et al., 2008). PD1 is highly expressed on Treg, and promotes Tregs proliferation in the presence of its ligands. Since many tumors have highly infiltrating Treg, blocking the PD-1 pathway may enhance the antitumor immune response by reducing the number or inhibiting the activity of Treg (Francisco et al., 2009). Two ligands of PD-1 are PD-L1 and PD-L2 (Tseng et al., 2001). On solid tumor cells, the main PD1 ligand expressed is PD-L1. Clinical evidence has been shown that PD-L1 expression by tumor cells suppresses local T-cell-mediated antitumor responses (Konishi et al., 2004). PD-1 monoclonal antibodies are currently commercialized and used clinically to treat several types of tumors (Pardoll, 2012; Wolchok et al., 2013; Larkin et al., 2015). The combination therapy strategy based on PD-1 inhibitors is expected to enhance the effect of PDT. Nanoparticles simultaneously encapsulated or loaded with photosensitizers and immune checkpoint inhibitors systematically exert antitumor immune efficacy. Choi et al. (2021) reported a nanoparticle (LT-NPs) that directly binds chemical drugs, photosensitizers and lysable peptide precursors. LT-NPs highly accumulate within tumor tissues *via* the EPR effect, and they are specifically cleaved to VPF and cathepsin B overexpression occurs in tumor cells, resulting in minimized side effects. At the same time, Because of LT-NPs containing PD-L1 blockers, the combinatorial treatment with anti-PD-L1 antibody not only leads to a powerful antitumor immune response but also efficiently inhibits the progression of distant pulmonary metastatic tumors (Figures 3D–K). However, it should be noted that due to the complex chemical synthesis process, it is difficult to control the quality of the functional nanoparticles in mass production. At the same time, the structure and function of nanoparticles need to be adjusted according to the specific clinical needs in the future study of PDT.

Indoleamine-2, 3-dioxygenase (IDO) is an immunomodulatory enzyme that is highly expressed in many types of solid tumors and catalyzes the oxidative metabolism of tryptophan to kynurenine (Feng et al., 2018). Deficiency of tryptophan or accumulation of kynurenine in the tumor microenvironment can inhibit T cell proliferation, promote Tregs production and activation (Awuah et al., 2015). It has been reported that some nanoparticles amplify PDT-induced immune responses by inhibiting IDO activity. Gao et al. (2020) investigated exfoliable prodrug vesicles in the tumor microenvironment in combination with a photosensitizer and IDO-1 inhibitor. Prodrug vesicles remain stable in the blood, avoiding drug leakage, and specifically accumulate at the tumor site. Meanwhile, the NLG919 prodrug was reactivated in the tumor microenvironment to inhibit IDO-1. Notably, the antitumor efficacy of the nanoparticles in the CT26 subcutaneous tumor model was relatively superior to that in the 4T1 tumor model, which may be due to more pronounced IDO-mediated immune evasion in CT26 tumors. It can be proved that in different tumor models, the inhibition of IDO shows different therapeutic effects, which has certain reference significance for the personalized adjustment of drug clinical application in the future (Gao et al., 2020).

## Conclusion

Numerous preclinical studies have shown that PDT is considered a promising strategy for the treatment of several types of tumors. PDT damages tumor cells and induces ICD to activates cellular subsequent immune response. As more has been learned about the immune regulatory mechanisms of PDT, PDT based on nano delivery systems are becoming the focus of clinical trials for cancer. According to the purpose and use of the treatment, nanoparticles are synthesized from different types of materials, such as lipids, polymers and inorganic compounds. Overcoming tumor-mediated immunosuppression by these nanocarriers *via* a combination or conjugation with immune checkpoint inhibitors would offer the possibility of amplifying antitumor immune responses and improving therapeutic efficacy. Other studies focused on induction of ICD, reprogramming of TAMs and regulating the tumor immune microenvironment by nanomedicine. Nevertheless, challenges and opportunities also remain for nanosystems driven photodynamic immunotherapy. Firstly, some nanopolymers and inorganic nanomaterials with poor biodegradability and biocompatibility significantly limits their clinical transformation. Optimized liposomes, extracellular vehicles, and biodegradable organic polymers etc. may be facilitate to clinical application (Cabeza et al., 2020; Pinto et al., 2021). Secondly, PDT initiated immune response and duration are relatively weak and short. There are still needed to optimize advanced biomaterials for eliciting effective immune responses and simultaneous activation of multiple immune signaling pathways. In addition to the combination of chemical drug and immune adjuvant to strengthen the host anti-tumor effect, the development of autologous tumor cell–based vaccines are emerging as a transformable and promising approach for personalized tumor therapy (Doix et al., 2019; Fang et al., 2020). Last but not least, more effective photosensitizers or nanocarriers is urgent to develop based on the further exploration of anti-tumor mechanism. Recently, PDT acts as a source of ROS is regarded to closely associated with a new form of regulated cell death, ferroptosis, which is characterized by the accumulation of iron-dependent ROS and lipid peroxides (LPO) to lethal levels. PDT can work in synergism with ferroptosis inducers and achieve a synergistic effect (Mishchenko et al., 2021). The combination action may lead to uncontrolled lipid ROS accumulation and cancer cell death by ferroptosis (Xu et al., 2020; Shui et al., 2021). More interestingly, PDT activates T lymphocytes to release IFN-γ which has been proved to downregulates both system xc^-^ subunits (SLC3A2 and SLC7A11) at the transcriptional level, thereby causing depletion of the intracellular GSH pool and triggering ferroptosis in cancer cells (Wang et al., 2019d; Zitvogel and Kroemer, 2019). The ferroptosis also induce tumor cells death through ICD, which is expected to continuously elicit and maintain the host’s anti-tumor immune effect after PDT. Therefore, ferroptosis induction in PDT is regarded as a powerful alternative strategy for reinforcing tumor therapeutic efficiency. It is clear that more work is required to develop novel integrated platform of PDT that induce prominent anti-tumor immunity effect in tumor microenvironment and limited toxic side effects in normal cells.
